# New Approach to Intelligence Screening for Children With Global Development Delay Using Eye-Tracking Technology: A Pilot Study

**DOI:** 10.3389/fneur.2021.723526

**Published:** 2021-11-03

**Authors:** Hong Xu, Xiaoyan Xuan, Li Zhang, Wenxin Zhang, Min Zhu, Xiaoke Zhao

**Affiliations:** Department of Rehabilitation, Children's Hospital of Nanjing Medical University, Nanjing, China

**Keywords:** eye-tracking technology, children, global development delay, cognitive assessment, cognitive development

## Abstract

**Objective:** There has become a consensus for detecting intellectual disability in its early stages and implementing effective intervention. However, there are many difficulties and limitations in the evaluation of intelligence-related scales in low-age children. Eye-tracking technology may effectively solve some of the pain points in the evaluation.

**Method:** We used an eye-tracking technology for cognitive assessment. The subjects looked at a series of task pictures and short videos, the fixation points of which were recorded by the eye-movement analyzer, and the data were statistically analyzed. A total of 120 children aged between 1.5 and 4 years participated in the study, including 60 typically developing children and 60 children with global development delay, all of whom were assessed via the Bayley scale, Peabody Picture Vocabulary Test (PPVT), and Gesell scale.

**Results:** Cognitive scores from eye-tracking technology are closely related to the scores of neuropsychological tests, which shows that the technique performs well as an early diagnostic test of children's intelligence.

**Conclusions:** The results show that children's cognitive development can be quickly screened using eye-tracking technology and that it can track quantitative intelligence scores and sensitively detect intellectual impairment.

## Introduction

Global development delay (GDD) refers to the developmental delay of children under 5 years of age in two or more developmental domains (1). This diagnosis is often used as a transitional diagnosis of intellectual disability (ID) in low-age children. The symptoms of ID present in infancy or early childhood (2), but it is difficult to accurately evaluate the intelligence level of children at these stages (3). ID can be diagnosed only when the child is over 5 years old and the cognitive ability is basically stable (4). However, the earlier GDD can be detected and intervention begun, the better the prognosis (4). This requires that children with GDD be identified using various technologies as frequently as possible.

It is widely recognized that early diagnosis and intervention can improve the prognosis for GDD (5). Identifying possible ID can help children and families acquire the appropriate services and support more quickly. It has long been recognized by scholars that early detection of ID facilitates timely intervention, maximizing the prognosis and preventing the occurrence of complications (5).

However, fewer than half of pediatricians in high-income countries use formal screening tools (6), and even fewer pediatricians do so in low- and middle-income countries (7). This is because it is a challenge to quickly and accurately assess the intelligence level of these GDD children with language development disorders or communication disorders. The common assessment tools, such as Bayley or Gesell scales take 30–60 min to complete, and it may take longer or be impossible to complete for children with poor communication attitudes. Scholars have made various efforts to improve this situation. When children cannot complete standardized tests, they use finger pointing, gaze, and partner-assisted scanning to perform cognitive assessments on children (8).

Eye-tracking technology has been applied increasingly more frequently in the field of psychological cognition since the introduction of the earliest diagnostic tools for such neurological diseases as oculomotor neuropathy and sleep disorders (9). Many studies prove that the direction of eye gaze reflects the focus of one's attention. Tracking and measuring eye movement can provide a non-invasive and rich indicator of brain function and cognition (10). In psychology, eye-tracking technology can help complete the measurement of memory ability (11) as well as understand the thought process behind learning, problem solving, and so on (10, 12). Assisting in early detection of autism spectrum disorder (ASD) can be achieved by observing reduced social attention in children via eye-tracking technology (13), and the cognitive level of patients with dementia can be evaluated using gaze-point data (14). Scholars have examined the feasibility of infrared eye tracking for assessing visual-orientation and sequence-learning abilities as well as attention to facial expressions in 9-month-old infants (15).

In this study, we designed a short and efficient cognitive evaluation tool based on eye tracking. It obtains eye-tracking cognitive scores through children's spontaneous gaze at a series of dynamic or static scenes on the display. These scenarios are composed of 13 tests, ranging from easy to difficult, and two short-term memory tests, which, respectively, investigate children's perceptual reasoning, verbal comprehension, short-term memory, and attention arranged in order from easy to difficult. The correct position of gaze should be set as the target image area on each test question. The eye-tracking cognitive score is obtained by calculating the sum of the time of gaze staying in the target image area. We measured eye-tracking scores in typically developing (TD) children and children with GDD and evaluated the correlation with a traditional cognitive assessment scale. The eye-tracking scores correlated well with the traditional assessment scores. It showed a good diagnostic performance in children with GDD.

## Methods

### Participants

At the Children's Hospital of Nanjing Medical University, 60 TD children and 60 children with GDD from 1.5 to 4 years old were recruited for physical examinations as outpatients. Before entering the main cohort, children were excluded after ophthalmologic examination because of oculomotor nerve movement disorders, visual impairment, or other diseases affecting eye movement. All the children were assessed by professional physicians using the Bayley scale, Peabody Picture Vocabulary Test (PPVT), and Gesell scale. The diagnosis of children with GDD was in accordance with the guidelines issued by the American Academy of Neurology (16). The scores of the Bayley cognition scale and Gesell adaptive behavior test for TD were all in the normal range, and the cases of brain injury, epilepsy, and hypothyroidism, which might cause intellectual impairment, were excluded.

Informed consent forms were signed by the legal guardians of all the children participating in this study. In addition, this study has been approved by the Ethics Committee of the Children's Hospital of Nanjing Medical University (batch Number: 202001001-1). The registration number in the Chinese Clinical Trial Registry is ChiCTR2000033524.

### Neuropsychological Assessments

The Bayley Scales of Infant Development Version 3 (17) are the most used standardized norm-referenced tool for identifying GDD, and it has been partly considered as the gold standard. The PPVT (18) is a brief and widely used standardized test of receptive vocabulary. Because of its non-verbal aspect, it is appropriate for children with expressive language impairment, and it is considered as a screening test of intelligence. There are five domains in the Chinese version of the Gesell Development Diagnosis Scale (19). All the children were assessed by professional physicians using the Bayley scale, PPVT, and Gesell scale.

### Experimental Procedures

#### Measurement of Gaze Points

Data concerning fixation duration were collected with the aSee Pro F140 desktop eye-movement analysis system produced by 7Invensun. The device does not require fixing the child's head to provide the refresh frequency of 140 hz and can accurately calculate the fixation point in the range of 50–95 cm.

**Figure 1:**
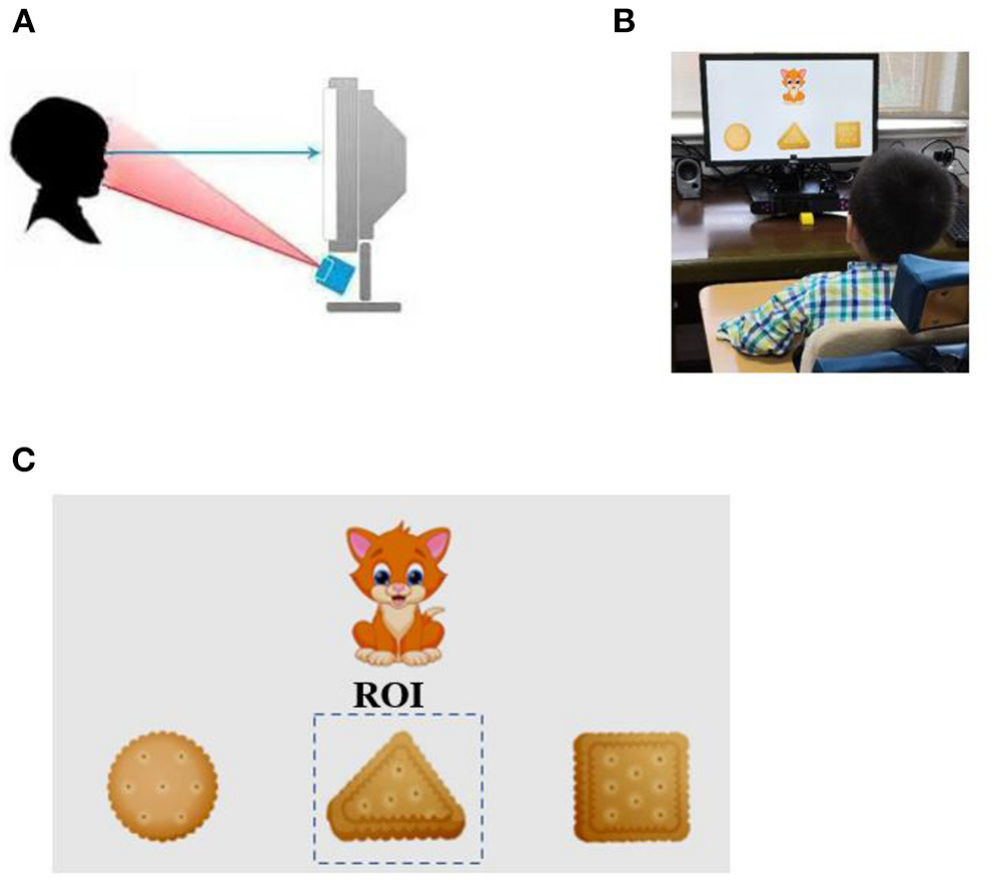
Rapid cognitive assessment using eye-tracking technology. **(A)** The eye-tracking system used in the study. The gaze point of the subject was recorded using infrared light source and camera located below the monitor. **(B)** Legend of eye-tracking test for children. **(C)** An example of a working task and representative gaze plot.

Children were seated in the gesture correction chair with a 21.5-in. display and eye-movement meter in front. First, an animation was played to attract the children's attention to the display, and then the test material was conducted formally after the necessary eye-movement meter calibration (Figures 1A,B). The test material consisted of 15 dynamic or static scenes, which were played sequentially and consecutively. The sum of the stay time of the children's fixation duration within the region of interest (ROI) was recorded (Figure 1C). The detailed procedure is described in Supplementary Information.

#### Cognitive Evaluation of Eye Tracking

1. Pay attention to the moving object: A small red ball rolls at a uniform speed from the left side of the screen and to the right side of the screen on a white background. The ROI is set as the moving red ball.
2. Preference for novelty: Based on task 1, a small blue ball is added, which rolls up and down in sync with the red ball. The ROI is set as the moving blue ball.
3. Look for objects/things that persist: An opaque tube is placed in the task 1 scene. The red ball rolls into the tube on the left and out the right. The ROI is set as the red ball's exit point on the right side of the tube.
4. Recognize color: The screen is divided into four quadrants (up, down, left, and right), and four fireworks tubes (red, green, yellow, and blue) appear in the quadrants, one in each quadrant. The audio recording “Red fireworks will be set off next” is played. The ROI is set as the red fireworks tube's quadrant.
5. Comprehend size: A cat appears at the top of the screen, a big fish on the bottom left, and a small fish on the bottom right. The audio recording “The cat eats small fish in the morning and big fish in the evening. Where is the big fish?” is played. The ROI is set as the big fish.
6. Understanding numbers: A cat appears at the top of the screen, one fish at the bottom left, and three fish at the bottom right. The audio recording “The cat eats one fish in the morning and three fish in the evening. Where is the single fish?” is played. The ROI is set as the single fish.
7. Comprehend shape: A cat appears at the top of the screen, and three cookies (square, triangular, and circular) appear below it. The audio recording “The cat is about to eat the triangular cookie” is played. The ROI is set as the triangular cookie.
8. Understand the name of item 1: The screen is divided into four quadrants with a fruit knife, soap, a cow, and a bus placed in each. The audio recording “The cow is going to turn into fireworks” is played. After a 5-s pause, the cow disappears and is replaced by fireworks. The ROI is set as the cow's quadrant.
9. Understand the name of item 2: The screen is divided into four quadrants with a pear, nail, ball of yarn, and leaves in each. The audio recording “The leaves are going to turn into fireworks” is played. After a 5-s pause, the leaves disappear and are replaced by fireworks. The ROI is set as the leaves' quadrant.
10. Understand the name of item 3: The screen is divided into four quadrants with a skirt, top, gloves, and a belt in each. The audio recording “The skirt is going to turn into fireworks” is played. After a 5-s pause, the skirt disappears and is replaced by fireworks. The ROI is set as the skirt's quadrant.
11. Understand the name of item 4: The screen is divided into four quadrants with bicycles, ambulances, tanks, and train carriages in each. The audio recording “The train carriages are going to change into fireworks” is played. After a 5-s pause, the train carriages disappear and are replaced by fireworks. The ROI is set as the train carriages' quadrant.
12. Understand the name of item 5: The screen is divided into four quadrants with a fencer, note-taker, speaker, and chef in each. The audio recording “The speaker is going to turn into fireworks” is played. After a 5-s pause the speaker disappears and is replaced by fireworks. The ROI is set as the speaker's quadrant.
13. Understand the name of item 6: The screen is divided into four quadrants with a cash register, fireplace, staircase, and washing table in each. The audio recording “The cash register is going to change into fireworks” is played. After a 5-s pause, the cash register disappears and is replaced by fireworks. The ROI is set as the cash register's quadrant.
14. Short-term memory 1: A light background frame is used to divide the positions of the cat, big fish, and small fish from task 5. The audio recording “Fireworks will be set off where the cat is now” is played. After a 5-s pause, fireworks are set off in place of the cat. The ROI is set as the area where the cat used to be.
15. Short-term memory 2: A light background frame is used to divide the positions of the cat, one fish, and three fish from task 6. The audio recording “Fireworks will be set off where the three fish are now” is played. After a 5-s pause, the fireworks are set off in place of the three fish. The ROI is set as the area where the three fish used to be.

The method for calculating eye-tracking cognitive scores is to use the sum of the fixation durations within the ROI for all tasks.

### Statistical Analysis

SPSS 22 and GraphPad Prism 5 software were used for statistical analysis. Spearman's rank correlation was used to calculate the correlation between eye-movement cognitive, Gesell, and Bayley cognitive scores. The diagnostic performance of the eye-movement cognitive and Gesell scores were determined by receiver operating characteristic (ROC) analysis. The area under the ROC curve was used as a diagnostic performance indicator to distinguish children with GDD from those that were TD. Results were expressed by mean ± standard, and *p* < 0.05 was considered to be significant.

## Results

### Participant Characteristics

General information about the participants was displayed in Table 1. TD and GDD were matched in terms of sex and age, so there was no significant difference between these two indicators (Table 1).

**Table 1:** General information of TD and GDD.

**Group**	**Number**	**Sex (M/F)**	**Age (months, $\bar{x}\pm$s)**
TD	60	32/28	36.43 ± 7.009
GDD	60	35/25	34.13 ± 8.062
*t*/χ^2^ value		0.304	1.668
*P*-value		0.581	0.098

### PPVT, Gesell, Bayley, and Eye-Tracking Technology in Different Groups

**Figure 2:**
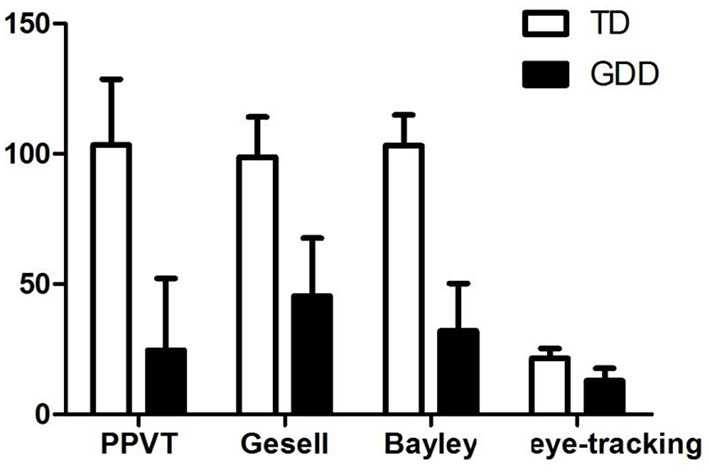
PPVT, Gesell, Bayley, and eye-tracking technology in TD and GDD.

All 120 children were assessed by professional physicians using the Bayley scale, PPVT, and Gesell scale. The scores of children with GDD were significantly lower than those of children with TD in the PPVT, Gesell, Bayley, and eye-tracking technology (*p* < 0.001) (Figure 2).

### Correlation With PPVT, Gesell, and Bayley

**Figure 3:**
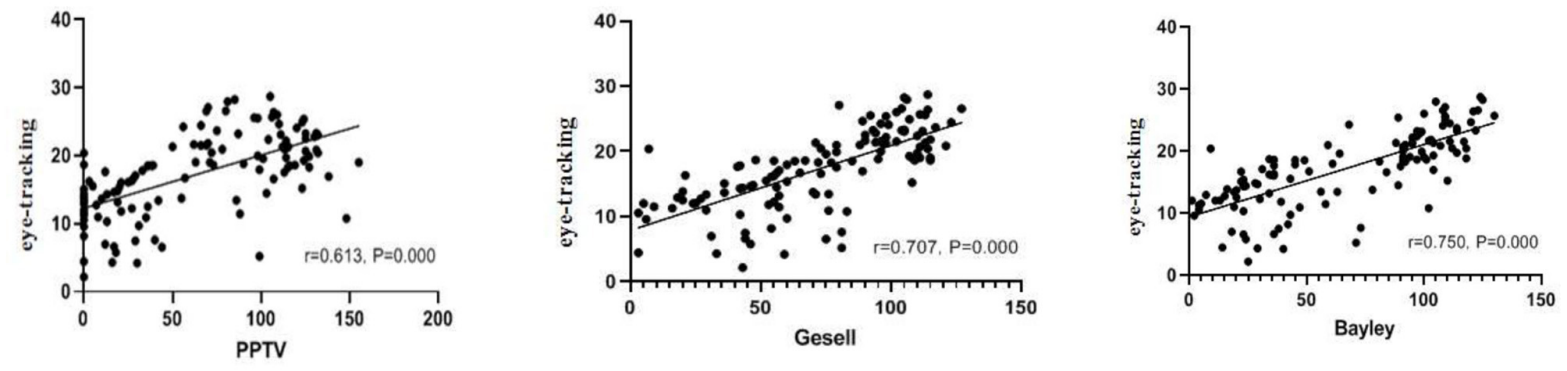
Correlation between PPVT, Gesell, Bayley, and eye-tracking technology.

Figure 3 shows scatter plots of eye-tracking technology with PPVT, Gesell scale, Bayley scale, respectively. The cognitive score of the eye-tracking technology was positively correlated with PPVT (*r* = 0.613, *p* < 0.0001), Gesell score (*r* = 0.707, *p* < 0.0001), and Bayley score (*r* = 0.750, *p* < 0.0001).

### Diagnostic Performance of the Eye-Tracking Technology

**Figure 4:**
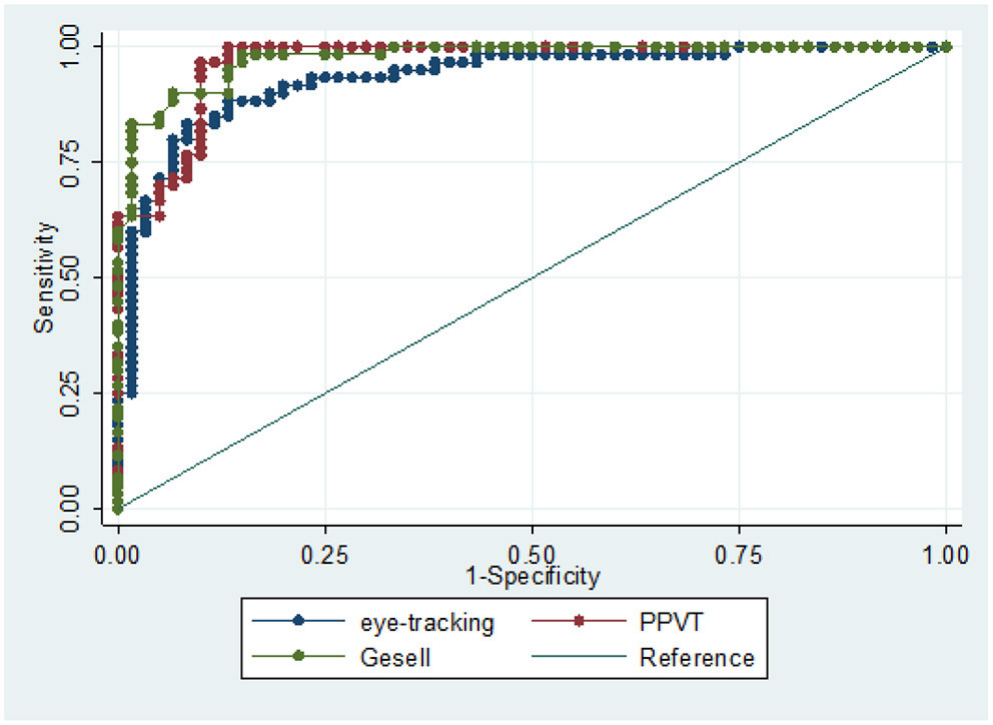
ROC curve analysis of diagnostic performance of the eye-tracking technology, PPTV, and Gesell scales to distinguish children with GDD from TD.

Diagnostic performance analysis of cognitive assessment based on eye-tracking is shown in Figure 4. The ROC curve analysis was used to assess the accuracy of eye-tracking technology for diagnosing children with GDD. To distinguish children with GDD from TD, eye-tracking technology archived an area under the curve (AUC) of 0.9331 (95% CI 0.889–0.977), which was comparable with the PPTV (AUC = 0.9669, 95% CI 0.940–0.994) and Gesell scale (AUC = 0.9756, 95% CI 0.954–0.996).

## Discussion

This study demonstrates that cognitive assessment based on eye tracking can be a practical method for detecting the cognitive level of children with GDD. By using eye-tracking technology in combination with short videos and images, the cognitive level of children can be quantitatively assessed. Cognitive level was also highly correlated with conventional neuropsychological tests, and the test was performed rapidly. The tasks used in this study were 15 dynamic or static scenarios. It includes children's perceptual reasoning (items 1–4), verbal comprehension (items 5–13), short-term memory (items 14–15), and attention arranged from easy to difficult. The results show that the score of the eye-tracking technology was significantly correlated with a variety of cognitive tests, such as Bayley scale, PPTV, and Gesell scale, which indicates that the cognitive score based on eye-tracking can reflect the overall cognitive function of children.

Using eye tracking to evaluate children's cognition does not need body movement and language expression, so it can overcome the requirements of conventional intelligence evaluation tools on children's cooperation and language expression ability. The cooperation level, motivation, and attention of ID children often affect the results of intelligence tests (20). This method is presented in the form of animations, and the test time is brief, so it effectively circumvents these uncertainties. It is especially difficult for children with ASD to complete intelligence tests because of social deficits when using conventional intelligence scales. Graphic test programs help maintain the attention of such children (21). In addition, the Bayley scale may underestimate the cognition of infants with motor retardation or physical disabilities because of the physical requirements of the test, such as manipulating building blocks (5). This method can be implemented only by maintaining the seat. Other advantages are that this program does not require highly qualified evaluators and can be carried out after simple training. At the same time, because the objective data are obtained by a computer, this evaluation process can be easily standardized, ensuring consistency of evaluation and repeatability of results.

Research demonstrates that a variety of gaze indicators obtained from eye-position data can be used to study the cognitive ability of adults (11). Eye-tracking measurements can provide insights into many aspects of cognition and cognitive development. In the cognitive study of infants, eye-tracking technology has been widely used to represent infants' expectations and cognitive processes (10, 22). Methods for measuring spontaneous orientation of stimuli (spontaneous scanning) are commonly used because such studies do not require children to perform specific eye-movement tasks, and the brain pathways that control reflex scanning are relatively mature at birth.

In recent years, eye-tracking technology has been used to learn the obstacle points for eye–hand coordination in children with cerebral palsy (23) and to assist in the rehabilitation of inpatients with tetraplegia (24). With its capacity to determine the relationships between language and the visual attention of developing children (25), eye-tracking technology shows that, in terms of time course for looking at a novel target image, there are differences between children who start speaking late and TD children; this is evinced by the former groups' difficulty in learning and understanding new words. Eye-tracking technology has also been used to detect abnormal visual preference for geometric images in children with ASD in its early stages, which is related to severity of symptoms and can be used as an early biomarker for ASD (13). In the evaluation of adult eye-tracking intelligence, Akane et al. developed video tasks, including deductive reasoning, working memory, attention, and memory, finding that cognitive scores based on eye tracking were closely related to scores of such neuropsychological tests, such as the Mini-Mental State Examination (14), which show good diagnostic performance in detecting patients with mild cognitive impairment and dementia. In this study, rapid and low-cost neurodevelopmental assessment of children was achieved by using eye-tracking technology.

However, cognitive assessment based on eye tracking depends on subjects' full visual function and requires listening to a sound cue, so it is not suitable for children with audiovisual impairment. These are the limitations of the method. At the same time, the program cannot fully cover all the dimensions of children's intelligence tests, such as operation and deduction, so it can not completely replace the conventional testing tools. However, this does not prevent the technology from being tailored to young GDD children. This technique has strong psychometric characteristics, considerably reduces the time involved in assessment, is easy to use, requires fewer participants, facilitates standardization, is reliable and effective, and possesses the basic elements of intelligence screening tools (26). Therefore, it can still be used as an effective screening tool for early identification of children with ID. Furthermore, children's age and stage can be subdivided, and their cognitive processes can be further evaluated by analyzing pupil dilation and spontaneous blink rate to further assess cognitive level (10).

## Conclusions

This study tested a novel approach for intelligence screening children with GDD. Assessing the cognitive level of children with GDD via the eye-tracking technology is highly correlated with conventional neuropsychological tests, obtains results rapidly, and performs well as an early diagnostic test of children's intelligence.

## Data Availability Statement

The raw data supporting the conclusions of this article will be made available by the authors, without undue reservation.

## Ethics Statement

The studies involving human participants were reviewed and approved by Ethical Commission of Children's Hospital of Nanjing Medical University (Batch Number: 202001001-1). The registration number in the Chinese Clinical Trial Registry is ChiCTR2000033524. Written informed consent to participate in this study was provided by the participants' legal guardian/next of kin.

## Author Contributions

XZ, HX, and XX conceptualized the idea and designed experiment. XX, LZ, WZ, and MZ contributed to clinical data collection and assessment. XZ and HX analyzed the results and wrote the manuscript. XZ approved the final manuscript. All authors read and approved the final manuscript.

## Funding

This work was supported by National Natural Science Foundation of China (81501946), Jiangsu Women and Children Health Association Scientific Research Foundation (FYX202013), and Nanjing Medical Science and Technology Development Foundation (YKK18144).

## Conflict of Interest

The authors declare that the research was conducted in the absence of any commercial or financial relationships that could be construed as a potential conflict of interest.

## Publisher's Note

All claims expressed in this article are solely those of the authors and do not necessarily represent those of their affiliated organizations, or those of the publisher, the editors and the reviewers. Any product that may be evaluated in this article, or claim that may be made by its manufacturer, is not guaranteed or endorsed by the publisher.
