# Supplementary material for: New Approach to Intelligence Screening for Children With Global Development Delay Using Eye-Tracking Technology: A Pilot Study
Source: Front Neurol. 2021 Nov 3;12:723526. doi: 10.3389/fneur.2021.723526 (PMC8595207; doi:10.3389/fneur.2021.723526)
Supplement: Supplementary file 1 [file Presentation_1.pdf]

Supplementary information

Task movie and picture for eye-tracking technology

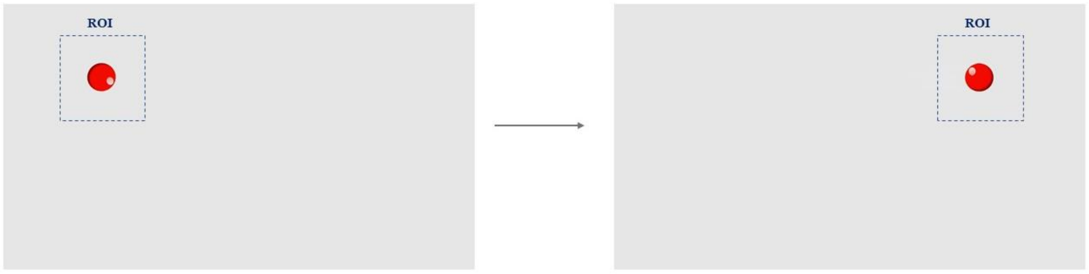

1. Pay attention to the moving object (9s in total, 5s in AOI)

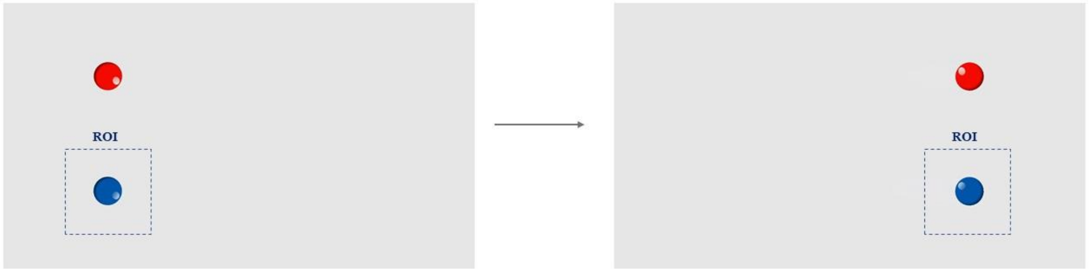

2. 2. Preference for novelty (9s in total, 5s in AOI)

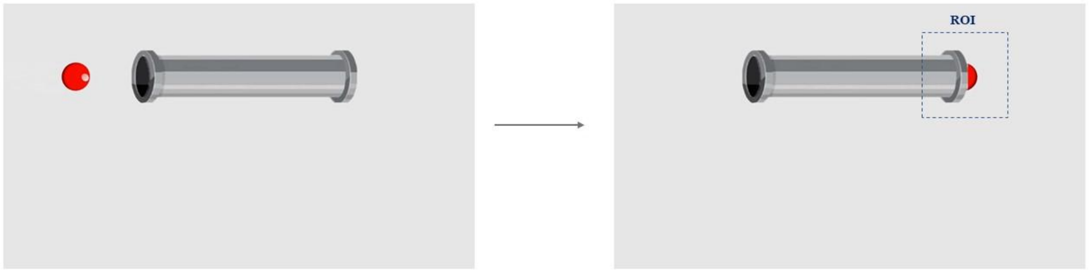

3. Look for objects/things that persist (9s in total, 2s in AOI)

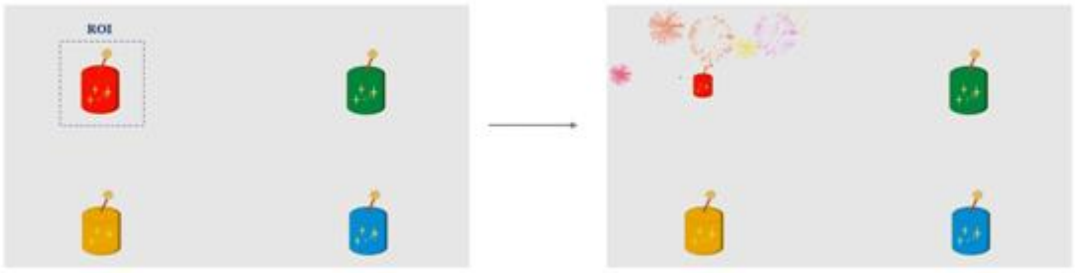

4. Recognize color (13s in total, 3s in AOI)

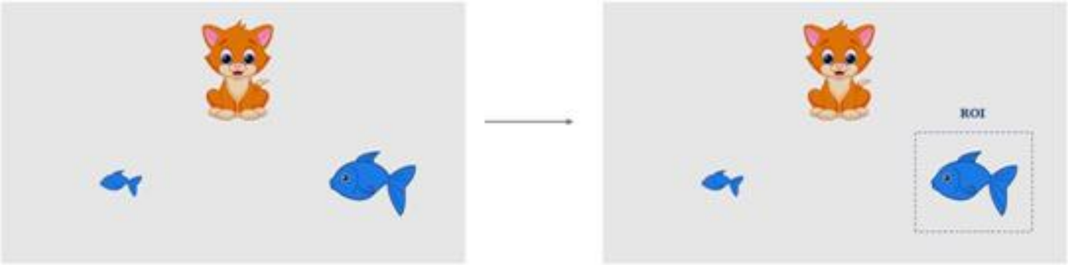

5. Comprehend size (12s in total, 3s in AOI)

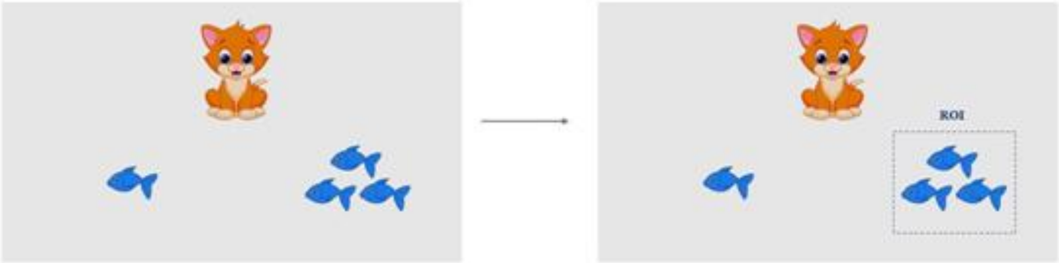

6. Understanding numbers (12s in total, 3s in AOI)

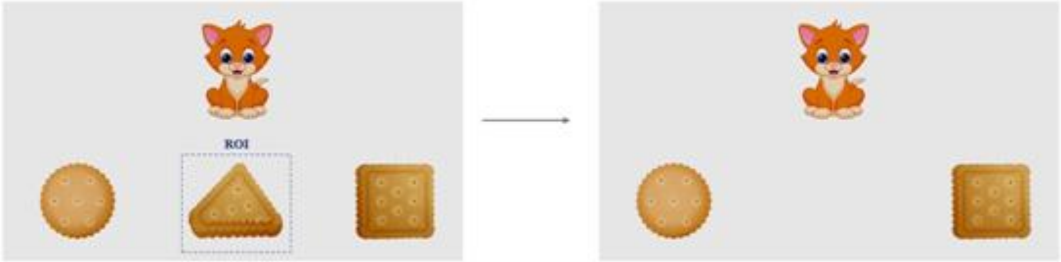

7. Comprehend shape (10s in total, 3s in AOI)

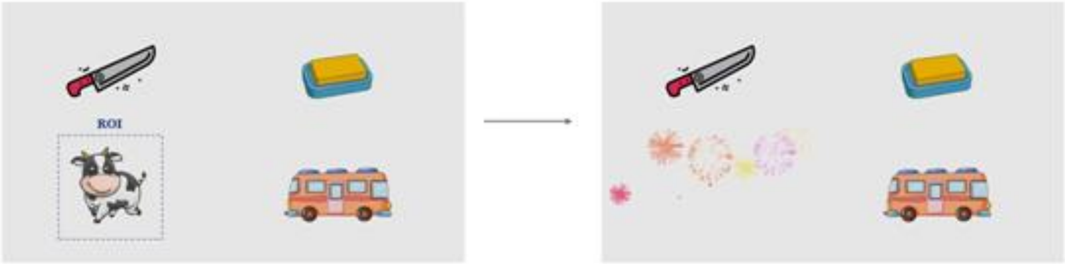

8. Understand the name of item 1 (11s in total, 3s in AOI)

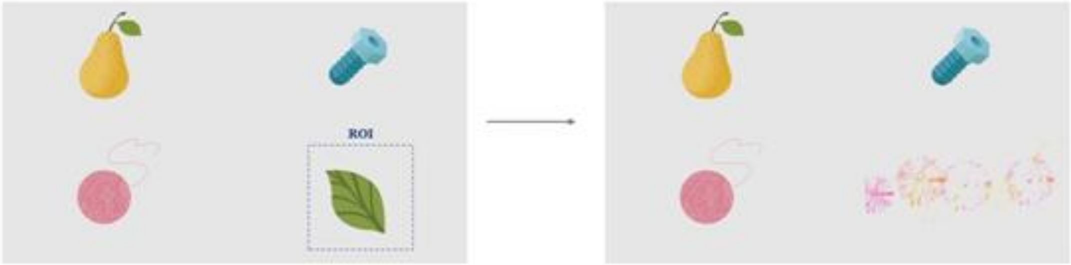

9. Understand the name of item 2 (12s in total, 3s in AOI)

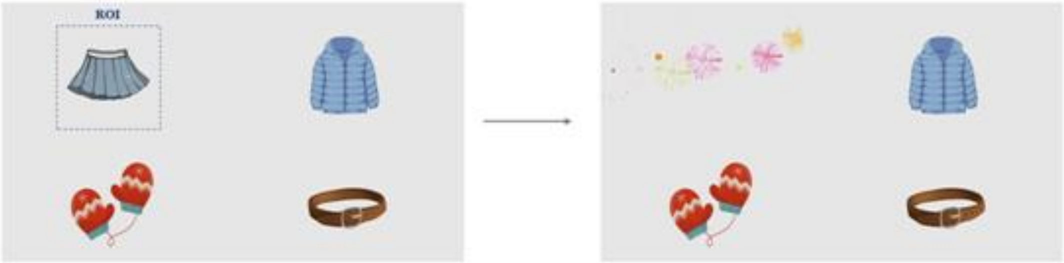

10. Understand the name of item 3 (12s in total, 3s in AOI)

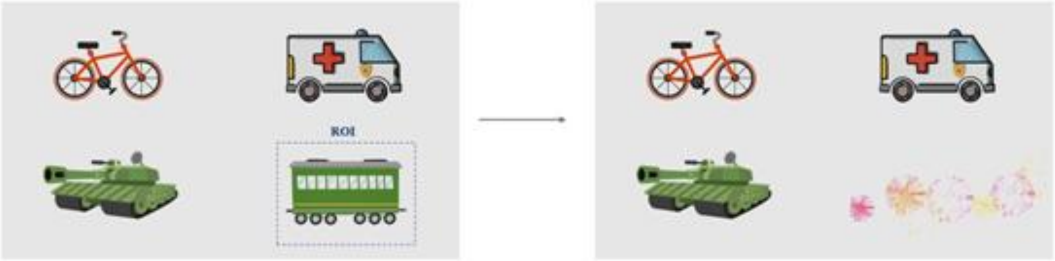

11. Understand the name of item 4 (12s in total, 3s in AOI)

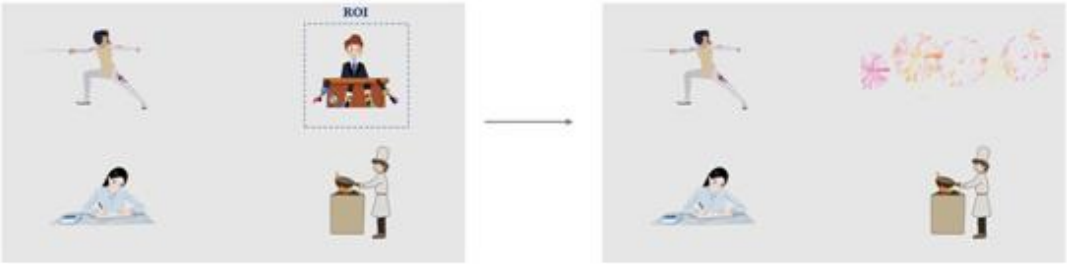

12. Understand the name of item 5 (13s in total, 3s in AOI)

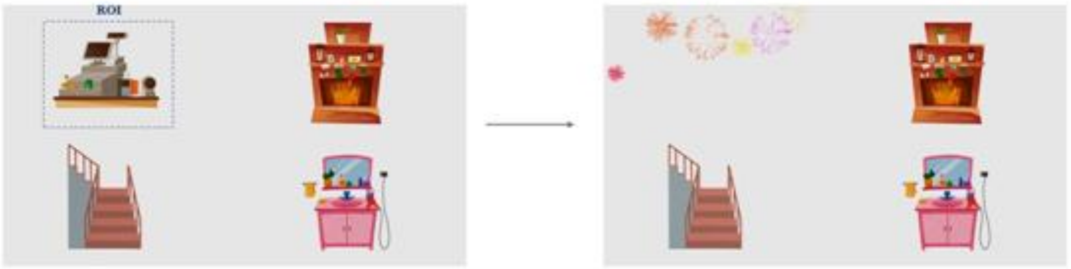

13. Understand the name of item 6 (12s in total, 3s in AOI)

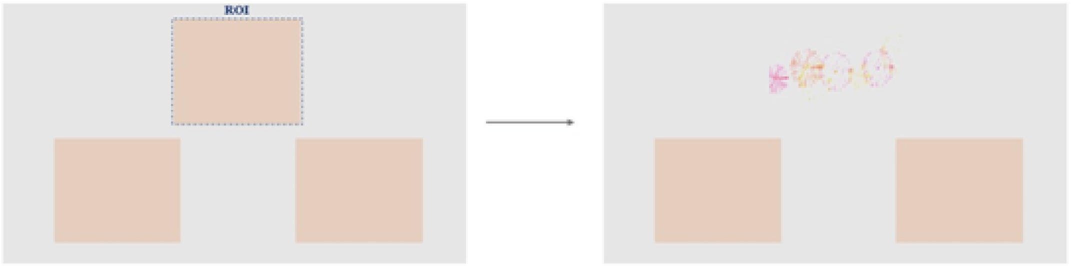

14. Short-term memory 1 (14s in total, 3s in AOI)

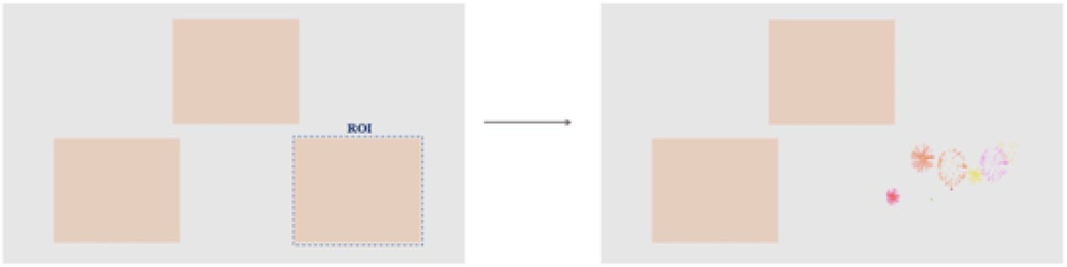

15. Short-term memory 2 (14s in total, 3s in AOI)
